# Concomitant Jejunoileal and Colonic Atresias

**Published:** 2012-04-01

**Authors:** Muhammad Riazulhaq, Elbaqir Elhassan, Diaa Eldin Mahdi, Adel Mutawalli

**Affiliations:** Department of Pediatric Surgery, King Faisal Hospital Taif, Saudi Arabia

**Dear Sir,**

Intestinal atresia is fourth common cause of neonatal intestinal obstruction [1]. Colonic atresia is relatively rare with an incidence of 1:40,000 to 1:60,000 live births. Coexisting jejunoileal and colonic atresias are scarcely described in literature [2,4,5].

A full term male baby, weighing 2.9 kg, presented to us with neonatal intestinal obstruction. A routine ante-natal ultrasound had shown dilated loops of bowel. The baby did not tolerate feeds and pass meconium rather developed progressive abdominal distension and bilious vomiting. Abdominal X-ray showed dilated bowel loops. A contrast enema showed micro colon and cut off sign at splenic flexure (Fig. 1). Our preoperative diagnosis was colonic atresia. After resuscitation and optimization, operation was performed that revealed concomitant type I jejunoileal and transverse colon atresias (Fig. 2,3). After excision of 10 cm of dilated proximal jejunum, an end-to-oblique jejunojejunal anastomosis was performed; divided colostomy was done at the level of colonic atresia. Post operative course was uneventful. Patient was discharged on 8th post operative day. The patient had a rectal biopsy done at 6th week follow-up. Biopsy showed presence of normal ganglion cells. Appointment was given for colostomy reversal.

**Figure:**
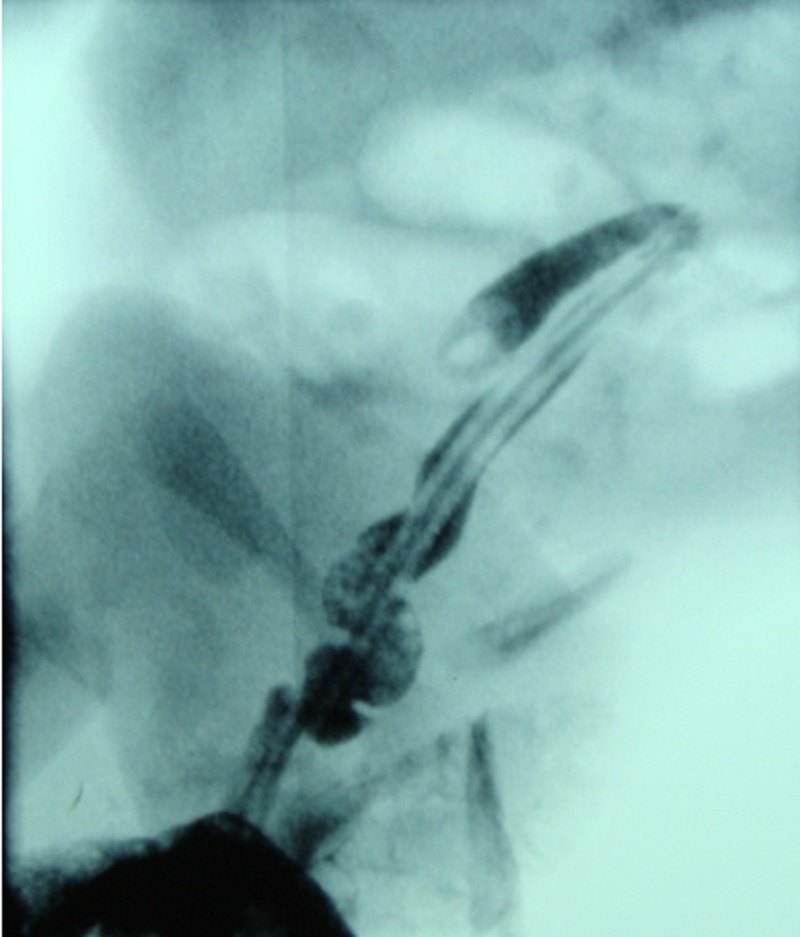
Figure 1: Microcolon.

**Figure:**
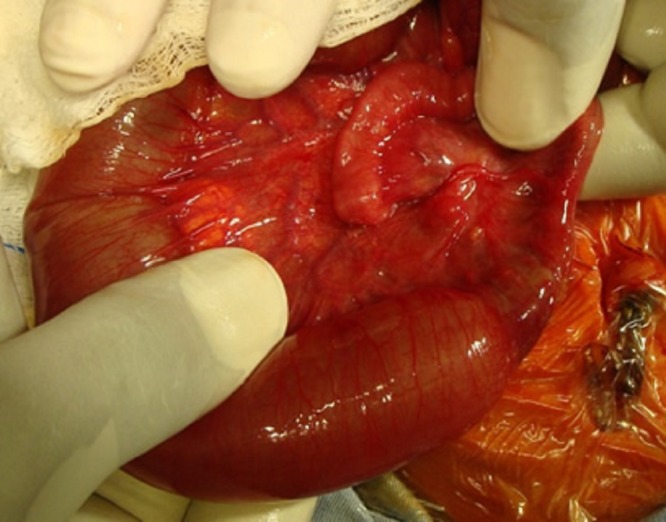
Figure 2: Type-I jejunal atresia.

**Figure:**
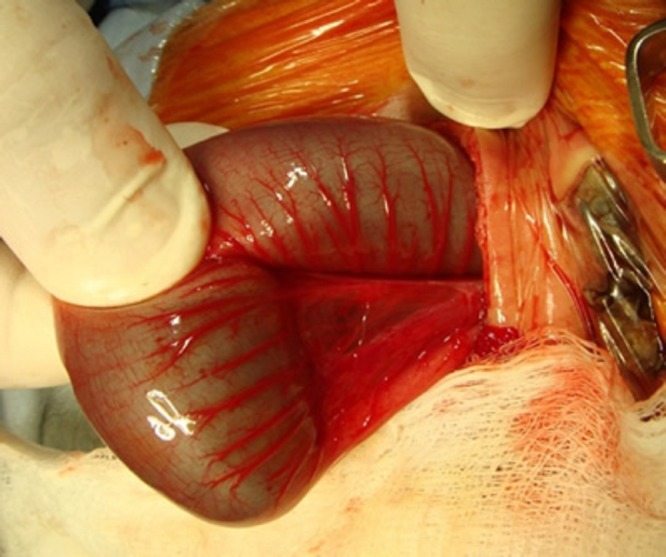
Figure 3: Type-I colonic atresia.

Barnard and Louw put forward vascular incidents as a probable etiology of intestinal atresias based upon their experiments on dog foetuses [3]. Nevertheless, failure of recanalization resulting in luminal mucosal webs in the jejunum and transverse colon seems a valid etiology in our case. Neonates with intestinal atresia present with a range of symptoms typical of intestinal obstruction. Contrast study showing a cutoff sign while transit through colon is highly suggestive of colonic atresia as documented in the index case.

Kernak et al mentioned 22 years experience on colonic atresia. They dealt 18 patients of colonic atresia while 4 were concomitant cases of jejunoileal and colonic atresia. [4]. DallaVecchia et al mentioned their 25 years experience on intestinal atresia. Out of 277 cases, only 5 were concomitant cases of jejunoileal and colonic atresia [5].

The surgical options as to concomitant jejunoileal and colonic atresias largely depend upon level of jejunoileal and colonic atresias. A primary anastomosis for jejunoileal atresias and a colostomy for colonic atresia is an acceptable option for proximal small bowel atresias. Ileostomy for distal ileal atresia and primary colocolic anastomosis is another option [4]. End ileostomy, Jejunocolic and ileocolic anastomosis are other options depending upon the length of bowel between both atresias.

## Footnotes

**Source of Support:** Nil

**Conflict of Interest:** None declared

